# The Roots of Diversity: Below Ground Species Richness and Rooting Distributions in a Tropical Forest Revealed by DNA Barcodes and Inverse Modeling

**DOI:** 10.1371/journal.pone.0024506

**Published:** 2011-09-19

**Authors:** F. Andrew Jones, David L. Erickson, Moises A. Bernal, Eldredge Bermingham, W. John Kress, Edward Allen Herre, Helene C. Muller-Landau, Benjamin L. Turner

**Affiliations:** 1 Smithsonian Tropical Research Institute, Balboa, Ancon, Republic of Panama; 2 Department of Botany, National Museum of Natural History, Smithsonian Institution, Washington, DC, United States of America; 3 Department of Marine Science, Marine Science Institute, University of Texas at Austin, Port Aransas, Texas, United States of America; Michigan State University, United States of America

## Abstract

**Background:**

Plants interact with each other, nutrients, and microbial communities in soils through extensive root networks. Understanding these below ground interactions has been difficult in natural systems, particularly those with high plant species diversity where morphological identification of fine roots is difficult. We combine DNA-based root identification with a DNA barcode database and above ground stem locations in a floristically diverse lowland tropical wet forest on Barro Colorado Island, Panama, where all trees and lianas >1 cm diameter have been mapped to investigate richness patterns below ground and model rooting distributions.

**Methodology/Principal Findings:**

DNA barcode loci, particularly the cpDNA locus *trnH-psba*, can be used to identify fine and small coarse roots to species. We recovered 33 species of roots from 117 fragments sequenced from 12 soil cores. Despite limited sampling, we recovered a high proportion of the known species in the focal hectare, representing approximately 14% of the measured woody plant richness. This high value is emphasized by the fact that we would need to sample on average 13 m^2^ at the seedling layer and 45 m^2^ for woody plants >1 cm diameter to obtain the same number of species above ground. Results from inverse models parameterized with the locations and sizes of adults and the species identifications of roots and sampling locations indicates a high potential for distal underground interactions among plants.

**Conclusions:**

DNA barcoding techniques coupled with modeling approaches should be broadly applicable to studying root distributions in any mapped vegetation plot. We discuss the implications of our results and outline how second-generation sequencing technology and environmental sampling can be combined to increase our understanding of how root distributions influence the potential for plant interactions in natural ecosystems.

## Introduction

Plant-soil and underground plant-plant interactions have direct implications for the conservation of biodiversity, plant productivity and the sequestration of carbon, and understanding of local ecosystem responses to global environmental change [1], [2]. However, the relative importance of competition for nutrients [3], [4], the effects of soil pathogens [5]–[7], and microbial mutualists including mycorrhizae [8] are poorly understood in natural communities despite the potentially large role they play in individual fitness, species coexistence, and ecosystem function. Studies of below ground processes in natural systems are currently limited by the difficulty of observing roots in situ and the lack of techniques to identify clearly where particular individuals or species forage and interact relative to the location of their above ground stems. Excavation of whole root systems has provided important insights into alternative plant foraging strategies [9], but it is inherently destructive and logistically difficult for plants with large root systems, such as trees, in natural systems. Large adult trees, which are key components in the biotic storage of carbon, are also not amenable to short-term experimental studies. Better methodologies are needed for assaying the degree to which plant species specialize on different resources (nutrients and water) both horizontally and vertically in the soil, how plant roots compete with each other for those resources, and how plants interact with soil microbial communities. Detailed information on below ground interactions will have profound implications for understanding species coexistence and ecosystem function [9], [10].

Detailed study, description, and mapping of below ground root networks would be possible if individual or mixed samples of roots taken from soil cores could be readily identified to the species or, ideally, the individual plant level. One promising approach to use DNA sequence data to identify roots to species either from single root fragments or from whole soil cores of roots composed of mixed samples of multiple species [11]–[17]. Direct sequencing of DNA from root fragments potentially offers the best way forward in diverse forests, given that a reference database of DNA sequences exists for co-occurring species [18]. However, even given an incomplete reference database, sequencing of commonly used genes such as *rbcL* might yield at least some indication of species identity given that the selected barcode locus can provide information on phylogenetic relatedness of coexisting species within a community [19], [20].

A global effort is underway to develop universally applicable DNA reference libraries composed of one or a few genes present in all organisms within a given taxon (termed DNA barcodes) that can provide species-level identification of samples [21]–[23]. DNA based identification techniques are poised to become a broadly applied method that can speed the process of species identification and aid in species discovery and delimitation [24]. DNA barcodes will also likely become important tools for ecological forensics, where sequence data can be applied to study cryptic ecological interactions within communities [25]. For example, recent efforts have revealed the utility of using DNA sequences for determining the plant species composition of vertebrate and invertebrate diets by extracting and sequencing plant DNA from animal guts, fecal samples, or honey, enabling the construction of more complete food webs [26]–[29].

A three locus DNA sequence reference library was completed recently for 296 species of trees and palms in the 50-hectare forest dynamics plot (FDP) on Barro Colorado Island (BCI), Panama [23], [30]. The library is composed of portions of the plastid coding regions ribulose bisphosphate carboxylase-Large subunit (*rbcL*), maturase K (*matK*), and the plastid intergenic spacer *trnH-psbA*. The first two have been recommended as the “universal barcode” for land plants [31]. For the BCI tree community, *matK* and *trnH-psbA* markers provide the most reliable diagnostic sequences in terms of correctly identifying samples to the level of species, with *rbcL* correctly discriminating among 70% of all species. Even though the reference library does not currently encompass all plant species present on the FDP, Kress *et al.* (2009) showed that each plastid region could correctly identify samples to the family level 100% of the time. This decreases the level of identification uncertainty when a sampled species is not included in the reference database (e.g. herbaceous plants or lianas are not yet included in the BCI DNA barcode database) and emphasizes the importance of using commonly sequenced regions for identification in concert with global sequence databases such as GenBank.

Here we describe the use of the BCI DNA barcode library to identify fine root fragments sampled from soil cores in a single hectare of lowland tropical rainforest from a mapped forest dynamics plot on BCI. We use the resulting data on species identity in concert with stem maps and inverse modeling of rooting extent to investigate three questions: 1) what are the levels of species richness within individual root cores? 2) What is the lateral extent of rooting distances within the samples? 3) Can we use inverse models to predict rooting extent as a function of neighborhood tree diameters and distances from the sampling point?

We find relatively high levels of species richness of roots within single cores given the small area and number of roots sampled, which indicates high potential for species overlap and competition below ground. Furthermore, we demonstrate the potential of inverse modeling techniques, originally developed for understanding spatial patterns of seed dispersal, to explore underground interactions among plants in fully mapped stands. Finally, we highlight the limitations of the approach taken here and discuss developing technologies that will enable broad-scale mapping of plant root networks and facilitate studies into their interaction with abiotic and biotic components of the rhizosphere.

## Materials and Methods

### Forest Dynamics Plot on Barro Colorado Island

We conducted our research within the 50-ha Forest Dynamics Plot (FDP) on BCI, Panama [32], in which all trees and liana stems ≥1 cm diameter at breast height (DBH) have been mapped, measured, and identified to species. The most recent tree census in 2005 found 300 tree species (http://ctfs.si.edu/datasets/bci/). The first liana census was completed in 2007 and found 163 species. In addition, a seedling census encompasses all individuals >20 cm height in a 1×1 m area in the center of every 5×5 m subplot [33].

**Figure 1:**
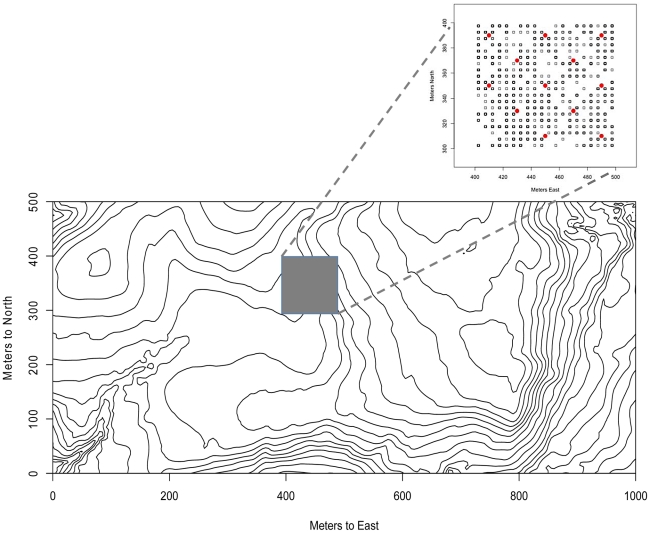
Map of sampled locations. Overview map of the forest dynamics plot on Barro Colorado Island and the focal hectare (inset) sampled in this study showing the locations of the soil cores (red circles) and 1 m^2^ seedling quadrats (open squares) where the quadrat had at least one seedling during the sampling period of 2006.

We examined root interactions in a randomly chosen 1-ha area of the FDP (Figure 1). The 2005 tree census found that this hectare contained 4023 individual trees >1 cm DBH representing 160 species. In addition to these mapped trees, a 2007 liana census revealed an additional 1022 individuals of 63 liana species in the focal hectare (S. Schnitzer, unpublished data). Of the 400 one-m^2^ seedling plots in this hectare, 302 had at least one seedling, and there was a total of 1596 individuals of 97 tree and 52 liana species in these plots. Mean seedling density in these plots was 3.99 individuals per m^2^ (4.54 SD) and mean species richness was 3.02 species per m^2^ (3.05 SD). Collectively, these censuses recorded 235 unique species of woody seedlings, shrubs, lianas, trees and palms in the focal hectare. Because seedling plots cover only 4% of the area and herbaceous plants are not censused, this is likely an underestimate of true vascular plant richness in the focal hectare.

### Soil and root sampling

Soil cores were taken from thirteen locations in the focal hectare (Figure 1). Cores were sampled using a 6.25 cm diameter auger. Each core was offset by 2.83 m in a northeasterly direction from the center of alternate 20×20 subplots. Surface cores were taken from each of the 13 sampling sites (0–10 cm and 10–20 cm samples), four of the 13 cores included additional sampling to 1 m (with samples separated into 0–10 cm, 10–20 cm, 20–50 cm and 50–100 cm sections) and one up to 3 m (separated as the 1 m cores and including 100–150 cm, 150–200 cm, 200–250 cm, and 250–300 cm sections, see Table 1 for maximum depth of each core). When coarse roots prevented coring, the sample was taken from a slightly different point to recover only fine and smaller coarse roots. Roots were removed by hand and dried in a desiccator with Dri-Rite desiccant (Chicago, IL). Samples were weighed to obtain the total “dry” mass of roots at each sample point. Samples were not oven dried as is typically done in root biomass studies because of the potentially negative effect oven drying could have on DNA quality.

**Table 1:** Species composition and abundance of roots within 12 soil cores determined by sequencing DNA barcode loci from the forest dynamics plot on Barro Colorado Island, Panama.

	Sampling Location												Above versus below ground abundance			
Species ID	A	B	C	D	E	F	G	H	I	J	K	L	sum root mass	Root mass rank	stem abundance rank	Stem basal area rank
*Alseis blackia*	1		5			2	5	6		1	1		0.364	3	8	11
Arecaceae *sp1*			1										0.040	14		
*Beilschmiedia pendula*						3			3			2	1.103	2	22	4
Bignoniaceae *sp1*											3		0.065	10		
Bignoniaceae *sp2*	1												0.001	28		
Bignoniaceae *sp3*											1		0.003	25		
*Brosimum alicastrum*			1					2					0.040	13	50	3
Burseraceae *sp1*								1					0.005	24		
Celastraceae *sp1*	1												0.013	19		
*Chrsophyllum sp1*								2				1	0.062	12		
*Chrysophyllum sp2*											1		0.118	6		
*Coccoloba sp1*											1		0.001	28		
*Coussarea curvigemmia*						1	2					1	0.219	5	19	65
Fabaceae *sp1*										1			0.001	28		
*Faramea occidentalis*						1							0.010	20	1	5
*Guapira standleya*						1							0.002	27	63	13
*Gustavia superba*								1				3	0.108	7	95	61
*Hybanthus prunifolius*		1						2					0.017	18	2	28
*Jacaranda copaia*			1										0.001	28	95	24
Malpighiaceae *sp1*									3				0.100	8		
*Pouteria reticulata*							1						0.006	22	50	22
*Protium tenuifolium*								1					0.001	32	20	19
*Quararibea asterolepis*	1	1			1	3		5	7	4			1.551	1	25	10
Sapindaceae *sp1*						1	4						0.029	16		
*Tabebuia guayacan*				1									0.020	17	95	21
*Taberemonta arborea*		2											0.063	11	26	7
*Tachigali versicolor*									3				0.009	21	34	27
*Tetragastris pamensis*			1										0.005	23	6	8
*Trichanthera gigantea*		1											0.029	15	130	99
*Trichilia tuberculata*			1	1						4	2		0.253	4	7	2
Ulmaceae *sp1*				1									0.003	26		
*Virola sebifera*								2					0.078	9	32	16
Fragments (N)	4	5	10	3	10	12	12	22	16	10	9	7				
Spp. richness	4	4	6	3	1	7	4	9	4	4	6	4				
Max Depth (cm)	20	20	100	100	20	300	20	100	100	20	20	20				
Total root mass (g)	2.34	1.49	0.73	6.10	2.64	1.82	1.23	3.58	4.30	1.72	2.56	5.74				
% mass sequenced	0.11	0.11	0.11	0.08	0.03	0.16	0.17	0.05	0.24	0.09	0.06	0.19				

Fragments are the number of fragments sequenced per core, spp. richness is the total number of species found from sequencing the core, total root mass is the total mass in the core, % mass sampled is the percent of the total root mass sampled that was successfully sequenced. Root mass rank is the ranking of the species according to the mass of successfully sequenced roots, stem abundance rank is the rank of the species in terms of number of stems in the focal hectare, and basal area rank is the ranking of the species according to basal area.

### DNA extraction

We sampled up to a maximum of 10 individual fine root fragments for each depth interval at each location (0–10 cm, 10–20 cm, etc, see above), measured the “dry” mass of each fragment, and placed 0.001 to 0.1 g of material from the individual root fragments into microcentrifuge tubes for DNA extraction. Root fragments with a mass greater than 0.1 g were included in the sample by cutting off a small portion of the root or by removing a portion of the cambial tissue in the case of small coarse roots. Tubes were submersed in liquid nitrogen and plates were disrupted using metal beads in a Qiagen mixer-mill. We used a combination of DNeasy Qaigen 96 plant kits and a modified CTAB extraction method in our DNA preparation [34]. Sequence recovery was greater using the modified CTAB method (results not shown).

### PCR conditions, sequencing, contig creation

We followed the procedure outlined in [23] for PCR amplification and sequencing of the *trnH-psbA* and *rbcL* markers. In brief, a single set of primers for each marker (see Kress et al. 2009 for primer sequences) was used and the same PCR reaction (2.0 µl 10× Biloine buffer, 0.8 µl 50 mM MgCl2, 0.8 µl 10 mM dNTP's, 1 µl each primer at 5 µM, 1 U taq, 1 µl DNA with H_2_O to 20 µl) and cycling conditions (95°C, 3 min 94°C-30 sec, 55°C-30 sec, 72°C-1 min) ×33 cycles, 72°C-10 min) were employed. Successful PCR were purified with ExoSap USB, Cat. # 78201) with 4 µl PCR mixed with 0.4 µl ExoSap and 1.6 µl H2O and incubated at 30°C for 30 min then 80°C for 20 min, and then 3 µl of the reaction mixture used directly in forward and reverse cycle sequencing reactions (95°C, 15 sec (95°C-15 sec, 50°C-15 sec, 60°C-4 min) ×30), which were then purified with Sephadex G50 and analyzed on an ABI 3730 capillary sequencer. Forward and reverse sequences were assembled in Sequencher 4.8 (GeneCodes) into contigs where discrepancies were edited and primer sequences trimmed. Following editing, sequences were exported in FASTA format for analysis with BLAST.

### Blast searches

Each new root fragment sequence was used in an all-against-all clustering analysis including all of the known sequences from the reference library [30] using the clustering method *blastclust* and default settings [35]. Root sequences were assigned to species or, in the case of ambiguous assignments, to genus based upon their clustering with a known species from the BCI sequence database and each other. In cases where roots did not cluster with a species within the BCI sequence database or where they clustered with multiple reference sequences (i.e. multiple species), sequences (both *psba-trnH* and *rbcL*) were then used in a MEGABLAST against GenBank and were assigned a genus or family based upon the closest hit (Table 1).

### Root distribution fits

We used inverse modeling to fit functions for the distribution of root mass relative to distances from potential source plants, in a manner analogous to approaches used to fit seed dispersal functions to seed trap data [36]–[38]. In our model, we treated different species the same as different genotypes of dispersed seeds were treated in Robledo-Arnuncio and Garcia [39] and Jones and Muller-Landau [40]. We refer to root mass here as the mass of roots within the sampled size classes because larger roots that would have prevented the soil coring from proceeding were not included; our estimates therefore omit larger main roots. We fitted data from only species identifications made in the top 20 cm, the depth to which data were available for the most points.

We fit a number of alternative models for root mass distribution (Appendix S1). The distribution was decomposed into the product of the total root mass (*M*), which we assumed scaled with tree diameter (*z*), and the probability density function for the distribution of root mass (*F*). We assumed total root mass scaled with trunk diameter as a power function, and fitted the scaling exponent (*β*). We assumed the probability density of root mass was a nonincreasing function of distance from the tree stem (*r*), and alternatively allowed the distance parameter of this density function to be a power function of trunk diameter (*αz^θ^*) or to be constant regardless of trunk diameter (*α*). Because relatively little is known about the probability distribution for encountering roots at different distances from the stem, we fit a wide variety of functional forms motivated by previous empirical [9], [41] and/or theoretical (e.g., [42]) work, including hyperbolic, inverse power, exponential, Gaussian, linear decline to zero at a threshold maximum distance, and constant to a threshold maximum distance. These are implicitly two-dimensional probability distributions; that is, they give the probability per unit area. Details of the fitting procedure are given in Appendix S1.

## Results

### Focal hectare

The focal hectare of this study had 4073 stems >1 cm in the 2005 census representing 165 species of trees, shrubs, and palms. The five most common trees in this plot were, in order of decreasing abundance, *Faramea occidentals* (Rubiaceae, n = 748), *Hybanthus prunifolius* (Violaceae, n = 550), *Desmopsis panamnesis* (Annonaceae, n = 259), *Mouriri myrtilloides* (Melastomataceae, n = 167), and *Hirtella triandra* (Chrysobalanaceae, n = 126). Of the 400 one m^2^ seedling plots, 302 contained at least one seedling >20 cm height in 2006. The most abundant seedlings on the plot were *Faramea occidentalis* (n = 146), *Hybanthus prunifolius* (n = 116), *Mouriri myrtilloides* (n = 97), *Eugenia oerstediana* (Myrtaceae, n = 93), and *Beilschmedia pendula* (Lauraceae, n = 81). The most abundant lianas on this focal hectare were *Cocoloba parimensis* (Polygonaceae, n = 197), *Doliocarpus olivaceus* (Dilleniaceae, n = 128), *Prionstemma corymbosa* (Hippocrataceae, n = 63), *Doliocarpus major* (Dilleniaceae, n = 50), and *Paragonia pyrimadata* (Bignoniaceae, n = 47).

### DNA sequence recovery

We successfully amplified 117 *trnH-psba* sequences from 288 individual root fragments from which DNA was extracted. One core had very few roots, none of which we recovered sequences from, so the final dataset includes samples from 12 of the 13 sampled locations (Figure 1). The number of root fragments successfully sequenced per sampling location ranged from 3 to 22 (mean ± SD 9.8±5.1) individual DNA sequences per core. These samples represented between 3 and 25% of the total root mass sampled in each core, with a mean of 12% of the root mass of the core included in our analysis (Table 1). For roots from which we recovered a *trnH-psba* sequence, we assigned 33 different species or higher taxon identities using *blastclust*, the BCI barcode database, and GenBank. We successfully amplified 89 sequences from 12 soil cores at *rbcLa*. Fragments were assigned to 33 different species or higher taxa. However, we unexpectedly amplified very few *matK* sequences from these samples and therefore do not report those results. Because of the limited resolving power of *rbcLa* sequence at the species level, [30], *rbcLa* sequences were used only to assign fragments to genera or families when a poor match for the *trnH-psba* sequence existed. Because *trnH-psba* allowed the best assignment to the species level [30], we focus on those results. The most common species identified in the *trnH-psbA* analysis was *Quararibaea asterolepsis* (Malvaceae, n = 31) distributed across eight of 12 cores, followed by *Alseis blackiana* (Rubiaceae, n = 19) in seven cores, *Belschmedia pendula* (Lauraceae, n = 8) in three cores, and *Trichilia tuberculata* (Meliaceae, n = 8) in four cores (Table 1). Roots indentified from soil cores are in contrast to common above ground stem abundance and relative basal area (Table 1).

### Richness within soil cores

We sampled all soil cores to a depth of 20 cm, so report those data first. Ninety root fragments were sequenced from top 20 cm of soil representing 29 different species (Table 1). Species richness in the surface 20 cm ranged between one and six species, with a mean of 3.66 species (SD = 1.66). The mean richness of the cores regardless of sample depth was 4.67 (SD = 2.06). The core with the greatest richness (9 species) was sampled to a depth of 1 m. We saw higher richness when a greater number of roots were sequenced within a sample, implying that we are underestimating true richness in many of our soil cores by not doing more complete sequencing of all roots in a core. For example, we sequenced roots representing only 5% of the total root mass that was sampled from the core with the greatest species richness. The deepest core for which individual roots were recovered (1.5 m) contained seven different species (Table 1). In the end, we assigned identity to 33 taxa across all depths representing approximately 14% of the measured plant richness (trees, seedlings, and lianas) in this focal hectare.

### Distance to nearest conspecific stem

**Figure 2:**
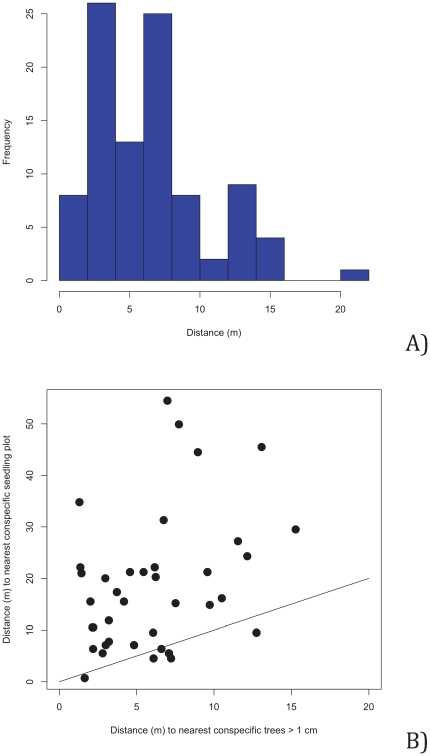
Distances from sampling point to nearest conspecific trees and seedlings. A) Distance to nearest conspecific individual >1 cm diameter for root fragments identified using *trnH-psbA* for samples on Barro Colorado Island. B) Relationship between sampling point for identified species and the nearest conspecific tree >1 cm diameter at breast height and the nearest conspecific seedling in a 1 m^2^ seedling plot. The line is the 1∶1 line.

**Figure 3:**
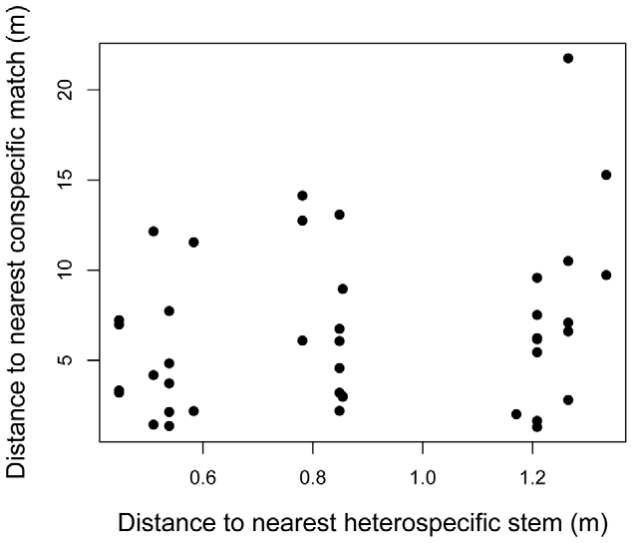
Distance from sampling point where a root was identified using DNA to the nearest stem (heterospecific or conspecific) versus distance to the nearest conspecific species matching the DNA sequence.

For the distance to nearest individual conspecific stem >1 cm diameter for root fragments (ignoring multiple instances of roots of the same species within the same core) we found a range of distances of 1.30 to 21.75 m, with a mean of 6.57 m (SD = 4.53) and a median of 6.09 m (Figure 2a). Distances to nearest conspecific seedling in a seedling plot revealed that most of the time (Figure 2b) the nearest tree >1 cm DBH was closer to the sampled root than the nearest seedling in a seedling plot. The nearest heterospecific individual >1 cm DBH to the sampling points was almost always located at a shorter distance than the nearest conspecific >1 cm DBH, with a range of 0.44 to 1.33 m, a mean distance of 0.87 m, and a median of 0.84 m (Figure 3). However, we cannot rule out the role of non-censused seedlings near our soil cores as a source for roots (discussed below). For samples with poor matches to the BCI database, and ignoring multiple instances of the same species of root within a core (n = 9), the nearest confamilial liana stem ranged from 1.4 to 25.0 m away, with a mean of 9.7 m and a median of 6.9 m. In contrast, when we examined the nearest confamilial trees for these same poorly resolved root samples, distances ranged from 1.3 to 64.2 m, the mean was 16.6 m and the median was 10.3 m.

### Root distribution fits

**Figure 4:**
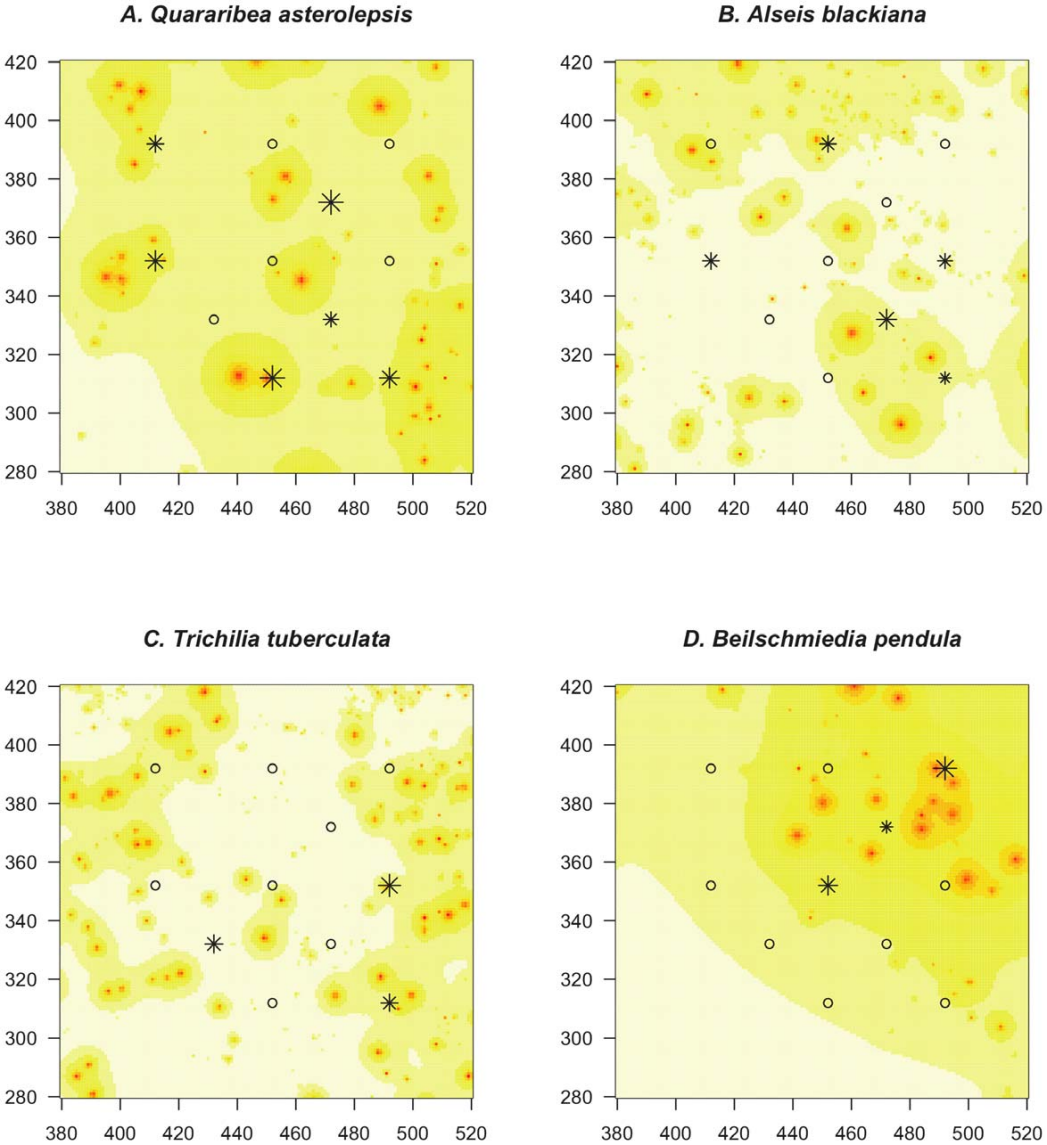
Maps of projected root distribution fits for four species. Map of the projected distribution of roots of four species in the top 20 cm of soil based on the rooting distribution parameter values that were fitted for all species combined. The root sampling points at which roots of the focal species were found are indicated with stars, with size scaling to the frequency of the species in proportion mass of samples genotyped. The root sampling points at which no roots of the focal species were found are indicated by open diamonds. The color shows the expected root density of the focal species under the best-fit model, with red indicating the highest value, yellow intermediate, and white lowest.

The model in which expected root mass is a hyperbolic function of distance from the stem provided the best fit to the dataset for data from the top 20 cm depths. This function assumes that root mass per unit area decreases as 1/distance, with no variation in the rate of decline depending on stem size. The fitted root mass scaling exponent (*β*) was 1.79, meaning the total root mass for these sized roots in the top 20 cm of soil increased with diameter to this power. The best-fit model explained 28% of the variation in the proportions of root mass of different species at different sampling points. The match between the predictions and observations was better for some species than for others, and there were systematic deviations from expected values in some species. The implications of the best-fit model for estimated spatial variation in root mass of a species across the plot are shown as maps of estimated root densities for the top four most abundant sampled species of roots (Figure 4).

## Discussion

Here we demonstrate that direct sequencing of individual fine roots at proposed DNA barcode loci, detailed stem maps, and inverse modeling of root distributions can be used to begin to dissect the organization of plant root networks below ground in a hyper-diverse lowland tropical moist forest. From root fragments randomly sampled from soil cores that covered less than a millionth of the area (0.037 m^2^ area or 0.096 m^3^ volume of soil) of the focal hectare in question, we recovered DNA sequence from roots representing at least 33 unique species or approximately 14% of the plant species richness in this hectare when considering all mapped and identified woody species (trees >1 cm, lianas, and seedlings). Below ground overlap among species in our system was high, with an average of 4.6 species in each soil core and a maximum of nine species of roots observed in a soil core taken to 1 m depth. The soil core with the most root fragments successfully sequenced (n = 22) was also the soil core that had the highest observed richness (9 species), implying that we have underestimated true root richness within all our soil cores by not sequencing all fragments present within our samples. Furthermore, incomplete sampling of soil depths and unaccounted for PCR failure due to species differences in DNA quality would also lead to underestimates of the true diversity of roots in the cores [17].

The limited scope of our sampling both in space and the number of DNA sequences recovered serves to highlight the potentially high overlap of species within individual soil core samples. Species overlap below ground at such a small scale is greater than one would expect based upon stem densities in the focal hectare. An equivalent area of at least 13 m^2^ (>350 times greater than our area sampled according to core diameter) and 45 m^2^ (>1200 times greater than our sampled area) on average would be required to sample the same number of species when counting stems of seedlings >20 cm height and trees >1 cm, respectively, in this hectare. Species accumulation curves for our root samples do not asymptote (data not shown), demonstrating that much larger samples of individual root sequences are required to adequately estimate below ground interactions in this and other diverse forests. This represents a challenge using the traditional Sanger sequencing and other directions are discussed below.

Evidence from BCI and other tropical forests suggests that much of the overlap that we see in our results is likely due to overlap of roots of large canopy trees that are included in the complete census rather than non-censused seedlings. There are several lines of evidence suggesting that most understory plants do not show as much overlap in their zone of influence as do large canopy trees. First of all, the density of plants in the understory is often quite low due to the shaded environment and the actions of herbivores and enemies in the understory [43]. Tropical tree seedlings have little overlap in their below ground zone of influence, suggesting that below ground competition among seedlings may be a weak force in determining seedling dynamics in the understory [43]–[45]. A survey of fine root biomass from the top 30 cm of soil on BCI found that fine root biomass averaged 372 g m^2^ [46], while entire root systems excavated for 48 understory shrub species averaged 34 g m^2^, implying that approximately 90% of fine roots in the upper 30 cm of soil volume are from canopy trees and lianas [47]. We expect even less root biomass from small seedlings than from understory shrubs. Nonetheless, seedlings may have contributed to our estimates here and need to be accounted for in modeling estimates in the future.

### Rooting distances and distribution fits

Another indication of the importance of large canopy trees in our results is that the relative abundance of roots in our samples is better predicted by the total basal area of a species within the hectare than relative stem abundance. The top 11 species in terms of basal area of trees >1 cm in this hectare includes the four most common species of roots identified in our sample ranked by the mass sampled, but including the 25 most common species of trees >1 cm in terms of stem number are required before the four most common species present in our soil cores are included. Our result contrasts with the results of Kesanakurti et al. [18], who found a strong correlation between above ground relative species abundances and below ground relative root abundances in a grassland community whose identity was determined using the DNA barcode *rbcL*. However, because of the several orders of magnitude greater variance in size and basal area in trees compared to grasses, one might expect a stronger correlation between above ground biomass and below ground biomass rather than stem number in tree communities.

The effect of basal area on expected root mass is also demonstrated by the results of our inverse modeling. The root distribution fits suggest that small root mass in the surface soil scales with stem diameter to the power 1.79, broadly consistent with previous studies based on excavating roots of individual trees. Our small root category includes both true fine roots (<2 mm in diameter) and smaller coarse roots. Functionally, fine roots are analogous to leaves in that both are resource-gathering organs, while coarse roots are analogous to stems. Fine root mass and leaf area are both expected to scale with the total cross-sectional area of the stem (i.e., diameter squared) according to the pipe model theory [48]. Coarse roots and stems make up the majority of below ground and above ground biomass, respectively, and previous studies have found that these scale with stem diameter to powers >2 in tropical forests: 2.59 for coarse roots [49], and 2.27–2.61 for stems [50].

We found that the mean distance from stem to the sampling location where individual fine roots had been identified was 6.21 m (SD = 4.1). The maximum distance we measured was 21.75 m from the sampling point to the nearest conspecific stem. Rooting extent in trees can be potentially large, which implies great potential for interaction among plants below ground relative to above ground structures. For example, Silman and Kisel [51] found that roots of *Ficus schultesii* at Cocha Cashu Peru extended between 7 and 103 m above ground, greatly exceeding the above ground canopy extent of individuals. Moreover, assignment of the nearest individual of a species as the source of a particular unknown root identified with DNA ignores the fact that roots may come from individuals located further away from the sampling point than the nearest individual of a species, which is certainly possible given the spectacular estimates in *Ficus*. This problem can potentially be ameliorated by using inverse modeling techniques to jointly account for the effects of distance and size on estimating root distributions.

The root distribution fits suggest that the total mass of such small surface roots declines hyperbolically with distance from stem, consistent with earlier models and fits of neighborhood interference [41], [52]. The residuals of the root distribution fits varied among species (data not shown), consistent with the presence of interspecific variation in rooting distributions not captured in our simple model. Larger datasets that sample across the landscape more intensively and that potentially identify all species present within a core will make it possible to quantify this interspecific variation, as well as test a broader range of more complex models that could potentially connect data on root mass, species identification, and nutrient concentrations. This will allow exploration of interactions below ground, specialization of species across nutrient gradients, leaf∶root∶shoot ratios, and tradeoffs among different species. Nevertheless, our results provide some of the first empirical data on the spatial extent of overlap of multiple species in a diverse tropical lowland rainforest and imply greater below versus above ground overlap. However, it remains to be seen if plants can avoid competition by hyperdispersion of roots that are capable of exploiting belowground areas with comparatively fewer neighbors [53].

### Future directions

It is clear that individual Sanger sequencing of root fragments will be prohibitively labor and cost intensive to get a representative sample from even a small area of a diverse tropical forest. Therefore, an important next step will be to combine root tissue samples from a core (an environmental sample) and recover sequences from all roots within the core simultaneously. Clearly, DNA pyrosequencing at one or more diagnostic loci is a promising way forward. Samples recovered from a single core could be PCR amplified without the need to separate individual roots and simultaneously multiplex 10s to 100s of sample cores in a single run. Second-generation pyrosequencing [54] will increase sample throughput and could also provide quantitative estimates of relative copy number and species content within each core, with obvious caveats. Although Sanger sequencing of individual roots recovered from cores is still relevant, the use of pyrosequencing and the simultaneous pooling of MID tagged samples from multiple cores will greatly accelerate our ability to diagnose species identities and therefore their interactions with other species. This approach is potentially applicable across all plant communities. Existing DNA sequence libraries as well as the continued development of global DNA barcode sequence databases should enable more detailed ecological studies of the spatial organization of root networks [18]. Another important step would be to combine sequencing of roots with information on soil nutrients and soil microbiota, including eubacteria, archea, and fungi, to more fully examine trophic interactions in soils. Such an approach will shed light on the importance of specific-specific host pathogens and mutualists including mycorrhizas, the distribution of plant roots relative to the abiotic environment, and reveal important niche axes for soil nutrients for individual species [55] .

We demonstrate the utility of a method that can be used to understand complex underground patterns of the organization of roots in hyper-diverse tropical forests, or any system of interest including experimental systems. Over a decade ago, a major research initiative was launched to explore the biological diversity of forest canopies. Elaborate systems have been devised and used to study and survey forest canopies at heights of up to 40 m, including a global network of canopy cranes [56], inflatable balloon platforms, and remote sensing airplanes and satellites. There is likely a far greater diversity of organisms beneath our feet much of it in association with plant roots. Soil root networks provide a critical, but poorly understood, link between above and below ground systems, so understanding these will reveal much about the functioning of ecosystems and provide insight into the mechanisms that maintain diversity in plant communities and the role they play in nutrient cycles and carbon sequestration.

## Supporting Information

Appendix S1 **Details of the root distribution fitting methods.**
(DOCX)
