# Supplementary material for: The Roots of Diversity: Below Ground Species Richness and Rooting Distributions in a Tropical Forest Revealed by DNA Barcodes and Inverse Modeling
Source: PLoS One. 2011 Sep 19;6(9):e24506. doi: 10.1371/journal.pone.0024506 (PMC3176281; doi:10.1371/journal.pone.0024506)
Supplement: Appendix S1 — Details of the root distribution fitting methods. (DOCX) [file pone.0024506.s001.docx]

To evaluate alternative models, we calculated the expected frequencies of every species’ root mass as a proportion of total root mass of genotyped species at every sampling point, and compared these with the observed frequencies. We evaluated models based on proportion of genotyped root mass associated with a species, rather than absolute mass of genotyped roots associated with a species, because the total mass of roots varied extensively among our sampling points, presumably due to high spatial heterogeneity in nutrient availability (Robledo-Arnuncio & Garcia 2007). Thus, , the expected proportion of genotyped root mass at sample point *j* that is of species *i*, is

where *N_i_* is the number of tree stems of species *i*, *z_k_* is the trunk diameter of individual stem *k*, *r_jk_* is the distance between sample point *j* and stem *k*, and *S* is the total number of tree species found as individuals of 10 mm or greater diameter in the study area. Tree stems were included if they were within 100 m of the sampling point.

The alterantive root distribution models that were fit are as follows. Throughout, is a normalization constant calculated so that the function integrates to 1 (specifically, ). Note that functions that are undefined at distance zero (hyperbolic, inverse power) are evaluated at distance m for all distances less than or equal to m. Further, stems are included as possible sources of roots only if they are within a distance m.

1. Hyperbolic decline with distance (note there are no fitted parameters for this model)

1. 2-D exponential decline with distance, with the speed of the decline scaling with tree size

1. 1-D exponential decline with distance, with the speed of the decline scaling with tree size,
2. Gaussian decline with distance, with the speed of the decline scaling with tree size,
3. ) Linear decline to a threshold distance that scales with tree size,
4. Constant root mass to a threshold distance that scales with tree size,

We searched for the parameter values and models that minimized the sum of squared deviations (least squares fit) for the proportion of genotyped root mass of species i at sample point j (summing over all I and j), . All analyses were done in R (2.10.1). Initial analyses revealed that fits were unstable when three parameters were fit simultaneously, and thus the parameter for the scaling of distance-dependence with tree size () was dropped. Of the remaining models, the hyperbolic model had the lowest sum of squared error, even though all other models had one additional parameter.
